# A high throughput serum bactericidal assay for antibodies to *Haemophilus influenzae* type b

**DOI:** 10.1186/s12879-016-1808-4

**Published:** 2016-09-05

**Authors:** Han Wool Kim, Kyung-Hyo Kim, JiHye Kim, Moon H. Nahm

**Affiliations:** 1 Center for Vaccine Evaluation and Study, Medical Research Institute, School of Medicine, Ewha Womans University, Seoul, Republic of Korea; 2 Department of Pediatrics, School of Medicine, Ewha Womans University, Seoul, Republic of Korea; 3 Department of Pathology and Microbiology, University of Alabama at Birmingham, Birmingham, AL USA

**Keywords:** *Haemophilus influenzae* type b, Serum bactericidal antibody assay, Haemophilus vaccines

## Abstract

**Background:**

The protective capacities of antibodies induced with *Haemophilus influenzae* type b (Hib) vaccines can be directly assessed in vitro with a Hib-specific serum bactericidal assay (SBA). However, the conventional SBA requires several tedious steps including manual counting of bacterial colonies, and therefore, it is seldom used.

**Methods:**

To overcome these limitations, we have improved the conventional SBA by using frozen target bacteria and by developing an automated colony counting method based on agar plates with the chromogenic dye 2, 3, 5-triphenyl tetrazolium chloride (TTC).

**Results:**

These changes enabled us to analyze about 100 serum samples per day per person by SBA. When the intra- and inter-assay precisions were studied, this assay showed a coefficient of variation (CV) ranging from 1 to 38 %. To monitor the long term assay stability for assays involving different bacteria lots, complement lots, and operators, we analyzed bactericidal indices of quality control samples obtained over a 6 year period and found the CV to be about 35–50 %. Lastly, our SBA results were compared with the ELISA results obtained using 90 serum samples from children. We showed that the bactericidal index correlated with IgG anti-Hib antibody levels (*r* = 0.84), with a bactericidal index of 10 corresponding approximately to 0.15 μg/mL IgG, the widely accepted protective level of antibody.

**Conclusion:**

We describe a simple high throughput SBA for anti-Hib antibodies that would be useful for evaluating various Hib vaccines. While additional work will be needed to standardize the assay, this SBA should greatly facilitate studies of Hib vaccines.

## Background

*Haemophilus influenzae* type b (Hib) was the leading cause of bacterial meningitis and a major cause of other serious invasive diseases among children aged < 5 years prior to the 1988 introduction of Hib conjugate vaccines [1, 2]. Hib conjugate vaccines have been found to be very safe and effective, and the use of the vaccines has reduced both the incidence of Hib diseases and the carriage and transmission of the organism in the community [2–5]. By 2013, Hib vaccines had been introduced into 189 countries [6].

To broadly deploy such a successful vaccine, substantial effort has been also made to include the Hib vaccine as a part of the combination vaccines [7]. Since different components in the combination vaccines may interfere with the Hib vaccine, these new Hib containing combination vaccines require assessment of the Hib component of the new vaccine formulation. To evaluate such combination vaccines, there is a persistent need for an anti-Hib assay.

The cases of invasive Hib in children increased in the United Kingdom when the Hib with diphtheria-tetanus-whole-cell pertussis vaccine (DTwP) was replaced with a diphtheria-tetanus-acellular pertussis (DTaP)-Hib vaccine. In their 2009 study, Kelly et al. found a higher antibody concentrations in children immunized in 1991 with Hib with DTwP than in children immunized in the late 1990s with DTaP-Hib [8]. Although the differences in the anti-Hib antibody titers between the two groups may be partly caused by reduced natural boosting opportunities after high coverage of Hib vaccine or use of concomitant meningococcal vaccine, this clearly demonstrated the need for monitoring anti-Hib antibody concentrations in the population in an active surveillance system. Moreover, various factors including the type of vaccine, immunization schedule, and ethnic differences could influence immune responses [9]. Therefore, anti-Hib assays for evaluating the immune response to Hib vaccines are required constantly.

Although the levels of antibodies to Hib can be easily measured with an enzyme-linked immunosorbent assay (ELISA), an assay capable of measuring the protective capacity of anti-Hib antibodies would be highly desirable. Since the primary protective mechanism against gram negative bacteria such as *H. influenzae* is antibody and complement-mediated bactericidal killing, a good surrogate assay for immune protection induced by Hib vaccines is an in vitro serum bactericidal assay (SBA) [10]. However, the conventional in vitro SBA is tedious to perform, mainly because counting colonies is so time consuming. Therefore, we have modified the conventional SBA by automating colony counting and miniaturizing the bacterial cultures required. Herein, we describe a new rapid SBA, its assay performance characteristics, and the correlation between the SBA and ELISA results.

## Methods

### Serum samples

Four quality control (QC) sera with very high (QCVH), high (QCH), medium (QCM), or low (QCL) titer sera prepared by mixing sera from 2 to 3 individuals (age range = 26 to 42 years) and were previously described [11, 12]. Their reference ranges of anti-Hib antibody titer were assigned after performing anti-Hib-antibody ELISA assay for more than 50 times [11]. Their reference ranges (mean ± standard deviation [SD]) were 43.00 ± 6.54 μg/mL, 4.38 ± 0.50 μg/mL, 1.52 ± 0.18 μg/mL, and 0.27 ± 0.07 μg/mL for QCVH, QCH, QCM, and QCL, respectively [11]. These sera were stored in 200-μL aliquots at −70 °C.

Ten pre-immune sera and 80 post-immune sera were selected based on their serum availability from a cohort of infants participating in an immunogenicity study of the Hib vaccine in Korean infants [12]. Anti-Hib IgG levels were previously determined for these residual sera [12] and 0.15 μg/mL was used as the lower limit of assay [11]. They were vaccinated with a single Hib vaccine (PRP-T or PRP-OMP).

### A high throughput SBA assay

SBA was performed as described [13] with modifications described below. All serum samples were heated at 56 °C for 30 min before testing was performed in duplicate. The heat inactivated sera were serially (three fold) diluted in a dilution buffer (Hanks’ buffer with Ca^2+^ and Mg^2+^ [Life Technologies, Grand Island, NY, USA] and 0.1 % gelatin). A frozen aliquot of Hib Eagan strain [14] was diluted in the dilution buffer to yield 750 colony forming unit (CFU) in 10-μL. Twenty μl of diluted serum was mixed with 10 μL of Hib solution and 10 μL of baby rabbit complement (Pel-Freez Biologicals, Brown Deer, WI, USA) in a microwell. The mixture was incubated for 2 min at 25 °C with shaking at 700 rpm and incubated for 30 min at 37 °C in a CO_2_ incubator without shaking. After stopping the reaction by placing the plates on ice for 15 min, 10 μL of the reaction mixture was plated on an approximately 1 cm by 3 cm area of a brain-heart-infusion (BHI) agar plate with 2 % Fildes enrichment (Becton Dickinson and Company, Sparks, MD, USA). After the fluid was absorbed into the agar, molten BHI agar (0.75 %) with Fildes enrichment (2 %) and 25 mg/L 2, 3, 5-triphenyl tetrazolium chloride (TTC; Sigma, St. Louis, MO, USA) was poured on top of the bottom agar layer.

The plates were incubated at 37 °C in a 5 % CO_2_ incubator for 16 h. Surviving bacterial colonies on the plates were counted with NICE (NIST [National Institute of Standards and Technology]’s Integrated Colony Enumerator); a free software [15] available from Dr. J. Whang in the US NIST) (http://www.nist.gov/pml/electromagnetics/grp05/nice.cfm). The colony counts were used to determine the serum bactericidal index (SBI). The SBI of a serum was defined as the dilution of the serum that results in half as many colonies as are seen with complement controls. If an undiluted serum sample killed 50 %, then the SBI is 4 in our system. To reduce the effect of variable activation of the alternative pathway on SBI result, each assay run included a complement control containing bacteria and baby rabbit complement with no serum. This complement control was used as 0 % killing in the SBI calculation. A control serum was included in each assay to monitor assay reproducibility. A detailed SBA method is posted on the following website: http://www.vaccine.uab.edu/.

### Reproducibility of SBA assays

The reproducibility of the Hib SBA was evaluated using the four QC sera, QCVH, QCH, QCM, and QCL. To determine intra-assay variability, sera were tested 5 times in one assay run. To determine short-term inter-assay variability, 11 independent assays using the same lot of reagents were performed but on different days.

To determine the effect of varying assay components, the assay was performed once with two different batches of bacterial stock and once with two different batches of complement. The overall mean ± SD and the coefficient of variation (CV) of SBIs were calculated.

To assess long term assay performance, SBA was performed with three QC sera (QCVH, QCH, and QCL) over a 6 year period and their bactericidal indices were plotted on a Levey-Jennings chart. During this period, the assay involved four different lots of baby rabbit complement, two different lots of Hib bacteria, and three assay operators.

### Statistical analysis

Correlations between SBAs were determined by Pearson’s correlation. Significant differences among assays were determined by Student’s *t* test. Comparisons between paired data were done by chi-square or Fisher’s two-tailed exact test. The significance level was set at a *P* value of <0.05.

### Ethics statement

The study protocol was approved by the Institutional Review Board of Ewha Womans University Hospital. The study was conducted in accordance with good clinical practices (national regulations and ICH E6) and the principles of the Helsinki Declaration. Informed written consent was obtained from all participants or their parents or legal guardians following a detailed explanation of the study.

## Results

### Establishment of the automated counting method

**Fig. 1:**
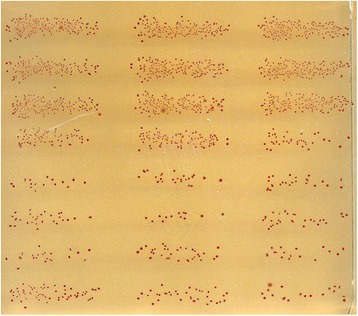
Micro-colonies of *Haemophilus influenzae* type b on a BHI agar plate with an agar overlay containing TTC. The average diameter of the micro-colonies was about 0.3 mm and the red colonies were distinct and could be readily counted with NICE

Automation of pneumococcal colony counting was attempted using an overlay of agar containing TTC [16]. However, TTC can be toxic to some bacteria resulting in the possibility that the overlay agar approach would not be feasible. Additionally, the overlay agar approach could have potentially failed because many bacteria may have simply detached from the base agar and floated over the overlay agar. Fortunately, Hib did not detach from the base agar and the overlay agar approach was determined to be feasible. To find a TTC concentration that gave optimal color without toxicity, we next investigated the effects of various concentrations (1–400 μg/mL) of TTC in the overlay agar. We found that the best results were obtained with 0.75 % agar containing 25 μg/mL TTC and 2 % Fildes enrichment [10]. The number of colonies observed with this overlay agar was equal to that of colonies observed without the overlay, indicating that the selected concentration of TTC was not toxic. With TTC, even very small colonies with a diameter less than 1 mm were clearly visible (Fig. 1), and allowed us to have up to 300 distinct colonies in a 1 cm by 3 cm area of an agar plate. In a conventional assay, a 96-well assay plate generates 96 agar plates. However, in our new procedure, a petri dish can have bacterial colonies from 48 reaction wells (i.e., half of the 96 well plate). Consequently, this allowed us to miniaturize the bacterial culture step and reduced the number of agar plates used for the assay by 48-fold.

**Fig. 2:**
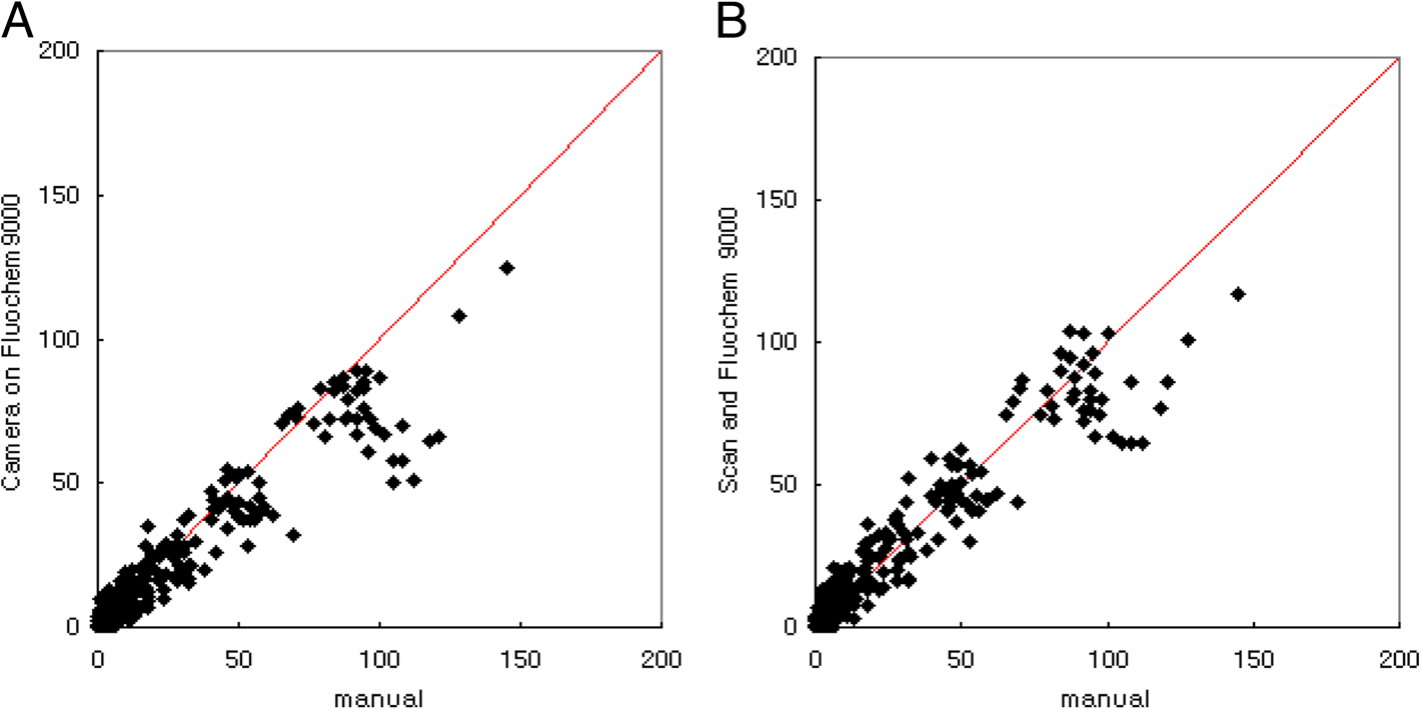
The numbers of colonies per spot obtained using a digital camera (Panel **a**) and a document scanner (Panel **b**) were compared with the true counts per spot (x-axis). The numbers of colonies were determined in 96 spots, and the true counts were established by manual counting. The lines indicate the best fit lines and their slopes were 0.84 and 0.89 for Panels **a** and **b**, respectively. The correlation coefficients (r) were 0.96 and 0.98 for Panels **a** and **b**, respectively. The coefficient was 0.99 for data points with less than 100 CFU/spot in both panels

To efficiently count colonies on the agar plates using the freely available program, NICE, we compared two different methods for acquiring images of the bacterial colonies on the petri-dish. We found that a camera can be used to obtain the images and that the colony counts obtained with digital camera images were highly correlated (*r* = 0.96) with the true manual counts as previously published [16], however, but the automated counts were slightly lower (by about 10–15 %) than the true counts (Fig. 2a). The deviation was noticeable when the number of colonies per spot exceeded 50. We subsequently found that images of four Petri dishes could be simultaneously obtained using a document scanner (Scanjet G4010, HP [Hewlett Packard Korea Ltd. Seoul, Korea]), which is readily available in many offices [15], and that the counts obtained with the scanner were highly correlated (*r* = 0.98) with the true counts. The slope of the best fit line was 0.89, suggesting that the colony counts determined with the scanner were slightly lower than the true counts.

### Reproducibility of Hib SBA assays

#### Intra-assay and inter-assay variability

The intra-assay (within-assay) variability of SBA was determined from 5 replicates of four QC sera performed in one assay run (Table 1). The inter-assay (between assays) variability of SBA was also determined on 11 runs carried out on different days (Table 1). Their CVs ranged 9–38 % for intra-assay variability and 31–36 % for inter-assay variability (Table 1). These CVs are comparable to CVs of a Hib ELISA (KHK unpublished observation).

**Table 1:** Precision of serum bactericidal assay with four quality control sera

		QCVH	QCH	QCM	QCL
		Serum bactericidal index			
Intra-assay variability (*N* = 5)	Mean	2876	1348	1865	824
	Standard deviation	252	191	333	314
	Coefficient of variation (%)	9	14	18	38
	Range	2507 ~ 3072	1118 ~ 1532	1554 ~ 2336	510 ~ 1260
Inter-assay variability (*N* = 11)	Mean	2841	1099	1017	637
	Standard deviation	1016	344	331	216
	Coefficient of variation (%)	36	31	33	34
	Range	1582 ~ 4376	538 ~ 1623	492 ~ 1444	514 ~ 1095

*N*, number

#### Long term assay variability

**Fig. 3:**
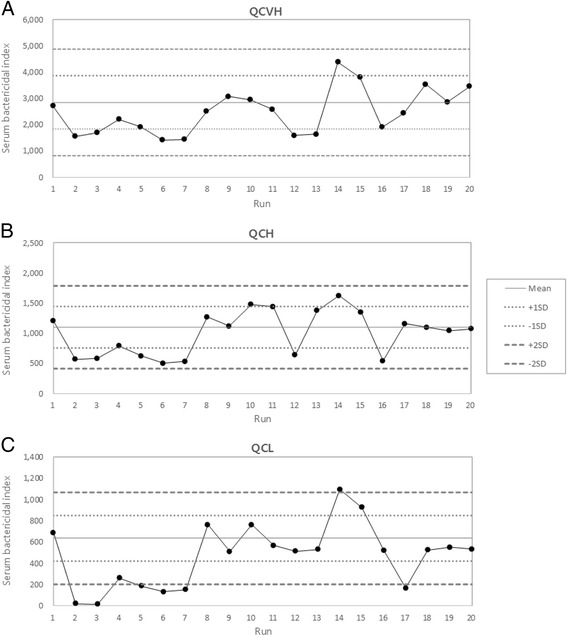
Levey-Jennings charts of serum bactericidal indices of three QC sera (**a** QCVH, **b** QCH, and **c** QCL) over 6 years. Runs 1–7 were performed in 2006, runs 8–15 in 2009, runs 16 and 17 in 2011, and runs 18–20 in 2012. The two dotted and dashed lines in each panel indicate one and two standard deviations (SDs) away from the mean, respectively

To investigate the long term reproducibility of SBA results, we obtained results from assays using three QC samples over a 6 year period (2006–2012) and plotted their SBIs in a Levey-Jennings chart. During this period, our SBA involved 2 different lots of target bacteria, 4 lots of complement, and 3 technicians. This chart therefore reflects the realistic variability in assay results and demonstrates that the SBA showed CVs ranging from 35 to 50 % (Fig. 3).

### Comparison of SBA with ELISA

**Fig. 4:**
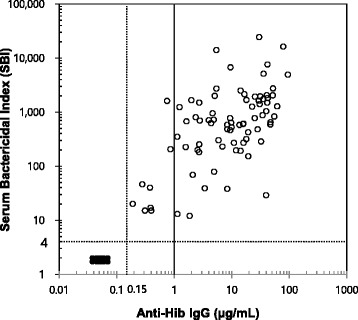
Comparison of SBA results with ELISA results. Ten sera with undetectable anti-Hib IgG (closed circles) and 80 sera with ≥ 0.15 μg/mL anti-Hib IgG (open circles) were tested. The anti-Hib IgG concentration (x axis) was compared with the serum bactericidal index, SBI (y axis). The vertical dashed line drawn at a concentration of 0.15 μg/mL anti-Hib IgG indicates the lower limit of the anti-Hib IgG ELISA and the horizontal dashed line at an SBI of 4 indicates the lower limit of the SBA. The vertical solid line drawn at a concentration of 1 μg/mL anti-Hib IgG is shown for comparison. The 10 data points (closed circles) with undetectable anti-Hib antibodies were assigned to have 0.075 μg/mL and 2 SBI for the purpose of statistical calculation

As anti-Hib antibody ELISA has been extensively used in studies of Hib vaccines and protective surrogates have been established for Hib antibody ELISA, it was important to compare our high throughput SBA results with the ELISA results. The SBI and the anti-Hib IgG concentrations are highly correlated (Fig. 4). The correlation coefficient (*r*) was 0.61 for the 80 post-immune sera and became 0.84 when all 90 samples were included. For the statistical analysis, it should be noted that we assigned an SBI of 2 and an anti-Hib antibody concentration of 0.075 μg/mL to the 10 pre-immune samples (Fig. 4). Interestingly, the 80 post-immune serum samples had anti-Hib IgG concentrations of greater than 0.15 μg/mL and, not surprisingly, all 80 samples had detectable SBI. In contrast, the 10 pre-immune samples contained 0.15 μg/mL anti-Hib IgG and all of them resulted in undetectable SBIs. Based on this data, we believe that 0.15 μg/mL and 1 μg/mL IgG anti-Hib antibody, the widely accepted protective levels, may correspond to SBIs of 10 and 64, respectively. Nevertheless, additional studies would be necessary to establish the corresponding cut off values.

## Discussion

Herein we have described a high throughput SBA that is easy to perform, requires no specialized equipment, and is relatively robust. One reason we developed this SBA as a high throughput was to accommodate the use of frozen bacterial aliquots, which eliminates the need to culture the target bacteria for each SBA experiment. Indeed, frozen aliquots of *S. pneumoniae* have been used extensively including for OPA. However, it was possible that Hib would not survive the freeze/thaw cycles as well as pneumococci do, since Gram negative bacteria are more fragile than Gram positives. However, frozen Hib aliquots were previously used for SBA by Romero-Steiner and colleagues [10] and we have also found that frozen aliquots of Hib strains can be used in SBA with little difficulty. Our experience should encourage the use of frozen aliquots for assays using other Gram negative bacteria. Indeed, we have found frozen bacteria can also be useful for Shigella SBA (unpublished information).

The primary reason for achieving a high throughput is the use of automated colony counting. Conventional SBAs require manual counting of bacterial colonies, which is tedious, time-consuming, and too labor-intensive for large SBA runs. To automate the counting process, we successfully adapted the simple overlay technique used for pneumococci [16] to Hib. We also adapted NICE, an open source program developed by the US NIST, to our SBA. Although NICE was designed to use a digital camera, we found a document scanner preferable because it did not require focusing and was simpler to set up. This extra simplicity has been highly useful to many investigators performing bacterial colony counting for cholera [17] and meningococci [18] cases.

Another major benefit of the overlay technique was that it allowed for miniaturization of bacterial colonies and a consequent reduction in the number of agar plates used for the experiment. Since Hib is a human pathogen, agar plates with Hib need to be autoclaved before they are discarded. To analyze ~100 samples, about 2,000 agar plates would have been needed in the absence of miniaturization and autoclaving 2,000 plates every day is not a simple task. However, the overlay technique reduced the necessary number of agar plates by 48-fold, reducing the number plates that require autoclaving to roughly 50 plates per day. In fact, the reduction in the number of plates needing to be autoclaved is another critical factor required to achieve a high throughput.

Our SBA, in addition to its high throughput, demonstrates stable analytical performance characteristics. It showed intra- and inter-assay precisions that were comparable to those of a Hib ELISA. Particularly interesting to us is that the SBIs of three QC sera obtained over 6 years showed CVs of 35–50 % in the absence of selection of complement lots or many other rigorous controls and standardization. Thus, while more rigorous evaluation of this assay is necessary, we believe that our SBA is robust and stable enough to be useful for vaccine studies.

In addition, we demonstrated that this high throughput assay compares well with ELISA results. Interestingly, the 10 sera with anti-Hib IgG concentrations <0.15 μg/mL resulted in undetectable SBIs whereas the 80 sera with concentrations of ≥ 0.15 μg/mL showed detectable SBIs. Our findings are consistent with the findings by Weinberg GA, et al., who reported the minimal anti-Hib IgG antibody concentration required to kill 50 % of Hib in vitro to be 0.22 μg/mL [19]. Taken together, we believe that SBA can complement the existing anti-Hib IgG ELISA in confirming an adequate immune response to the Hib vaccine. However, additional studies should be done to compare SBA with ELISA using many more samples [20].

## Conclusions

We describe a simple and high throughput SBA for anti-Hib antibodies that is easy to implement and practical for studying various Hib vaccines. Pneumococcal vaccine studies have clearly shown the importance of measuring the function of pneumococcal antibodies [21, 22]. Since ineffective anti-Hib antibodies exist [23, 24], an SBA for Hib antibodies will be needed to study new Hib-containing combination vaccines. While additional work (e.g., standardization) needs to be performed to refine our SBA, due to its simplicity and high throughput, should be very useful for future Hib vaccine studies.

## Abbreviations

BHI: Brain-heart-infusion
CFU: Colony forming unit
CV: Coefficient of variation
DTaP: Diphtheria-tetanus-acellular pertussis vaccine
DTwP: Diphtheria-tetanus-whole-cell pertussis vaccine
ELISA: Enzyme-linked immunosorbent assay
Hib: *Haemophilus influenzae* type b
NICE: National Institute of Standards and Technology’s Integrated Colony Enumerator
NIST: National Institute of Standards and Technology
QC: Quality control
QCH: Quality control sera with high titer sera
QCL: Quality control sera with low titer sera
QCM: Quality control sera with medium titer sera
QCVH: Quality control sera with very high titer sera
SBA: Serum bactericidal assay
SBI: Serum bactericidal index
SD: Standard deviation
TTC: 2, 3, 5-triphenyl tetrazolium chloride
